# A Case of Acute Kidney Injury, Proteinuria, and Thrombotic Microangiopathy Associated With Sunitinib Therapy in Metastatic Pancreatic Neuroendocrine Tumor

**DOI:** 10.7759/cureus.56660

**Published:** 2024-03-21

**Authors:** Lawanya Singh, Daniel Matassa, Sharon Li

**Affiliations:** 1 Internal Medicine, Rutgers New Jersey Medical School, Newark, USA; 2 Internal Medicine, University Hospital - Rutgers New Jersey Medical School, Newark, USA; 3 Hematology and Medical Oncology, Rutgers Cancer Institute of New Jersey, Newark, USA

**Keywords:** tyrosine kinase inhibitors (tki), tki, nephrotic syndrome, oncology, hematology, neuro-pancreatic syndromes, imatinib and sunitinib

## Abstract

There have been rare reports of patients developing nephrotic syndrome and thrombotic microangiopathy (TMA) with tyrosine kinase inhibitors (TKIs). We present the case of a patient with a history of metastatic pancreatic neuroendocrine tumor (pNET), treated with sunitinib, who rapidly developed TMA and acute kidney injury. The patient was successfully treated with cessation of sunitinib and administration of steroids. This case report contributes to the growing body of literature surrounding the rare side effects of TKIs and our experience with the management of these adverse events.

## Introduction

Sunitinib is a tyrosine kinase inhibitor (TKI) that targets multiple receptors, including vascular endothelial growth factor (VEGF) and platelet-derived growth factor (PDGF). Inhibition of VEGF most commonly causes hypertension and proteinuria, but there have been rare reports of patients developing nephrotic syndrome and thrombotic microangiopathy (TMA) [1,2]. We present the case of a patient with metastatic pancreatic neuroendocrine tumor (pNET) treated with sunitinib who had an initial response to treatment, but after a treatment holiday, on re-challenge, developed severe generalized anasarca, hypoalbuminemia, anemia, thrombocytopenia, proteinuria, and acute kidney injury within the course of two weeks. Symptoms resolved with cessation of sunitinib and high-dose steroids. These findings raised concern for sunitinib-related TMA, highlighting the need for clinicians to be astute to the potential toxicities associated with VEGF inhibitors.

## Case presentation

**Figure 1:**
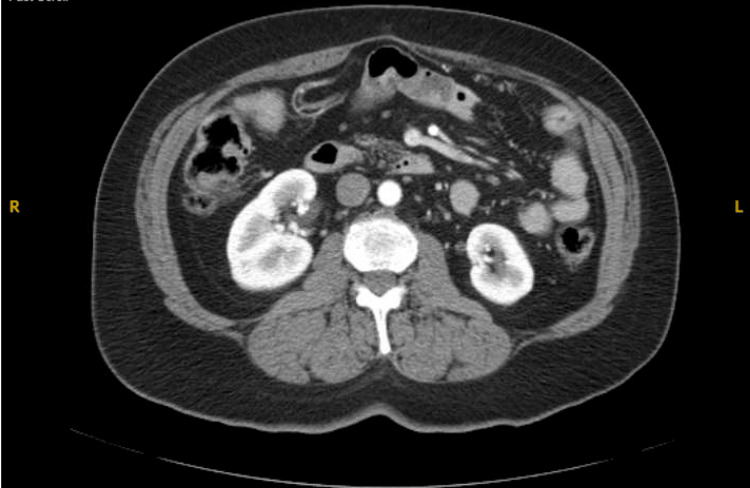
CT scan of chest/abdomen/pelvis CT, computed tomography

A 61-year-old female, with a past medical history of hypertension and hyperlipidemia, was diagnosed with stage IV pancreatic neuroendocrine carcinoma with peritoneal and liver metastases. Initial pathology demonstrated a well-differentiated 3 cm neuroendocrine tumor, grade 2 with extension to the posterior margin, Ki 67 10%, with metastases to the peritoneum. As per treatment guidelines for pNET, the patient underwent subtotal pancreatectomy, splenectomy, peritonectomy, and fulguration of peritoneal tumor implants [3]. The patient developed insulin-dependent diabetes mellitus, secondary to pancreatectomy. Postsurgery, the patient was started on octreotide and sunitinib given her metastatic disease. She remained on this for approximately one year. Of note, the patient encountered some hypoglycemic issues with sunitinib; re-staging scans showed no evidence of disease, so one year after initiation of treatment, she decided to stop treatment with sunitinib. Ongoing insurance issues resulted in intermittent follow-up. When she re-established stable follow-up, she was resumed on octreotide. She did well until re-staging. At this time, the patient reported increasing abdominal pain and discomfort. Computed tomography (CT) scan of the chest/abdomen/pelvis, which was approximately four years from surgery, showed increasing omental and peritoneal deposits (Figure 1). Due to progressive disease and onset of symptoms despite treatment with octreotide, the patient was resumed on sunitinib given her history of previous response.

One month after taking sunitinib, the patient presented to the hospital with progressively increasing diffuse edema and fatigue. The patient noted shortness of breath and chest tightness, as well as new swelling. Physical examination revealed a blood pressure of 166/78 mmHg, diffuse anasarca, bibasilar crackles, and left submandibular lymphadenopathy. Initial laboratory studies are detailed in Table 1.

**Table 1:** Initial laboratory studies WBC, white blood count; LDH, lactate dehydrogenase

	Result	Reference range
WBC	7.1x10^3^/μL	4.0-11.0x10^3^/μL
Hemoglobin	10.3 g/dL	12.0-16.0 g/dL
Hematocrit	30.1%	36.0-48.0 %
Platelet	130x10^3^/μL	150-450x10^3^/μL
MCV	95.3 fL	80.0-99.0 fl
Sodium	143 mEq/L	133-145 mEq/L
Chloride	114 mEq/L	97-110 mEq/L
Bicarbonate	21 mEq/L	23-30 mEq/L
BUN	24 mg/dL	8-23 mg/dL
Creatinine	1.4 mg/dL	0.5-1.0 mg/dL
Total protein	5.8 gm/dL	6.0-8.3 gm/dL
Albumin	2.9 gm/dL	3.5-5.2 gm/dL
LDH	712 U/L	120-246 U/L

The thrombocytopenia, elevated creatinine, and decrease in total protein and albumin were new for the patient. Peripheral blood smear showed few schistocytes. Sunitinib was discontinued and the patient was empirically started on IV methylprednisolone 1 mg/kg for possible nephrotic syndrome. The hospital course was also complicated by suppurative lymphadenitis of the jaw seen on physical exam and CT scan on admission, for which she was started on intravenous vancomycin 15 mg/kg every 24 hours IV, cefepime 2 g every 12 hours IV, and metronidazole 500 mg every eight hours IV. On day four of hospitalization, the patient had episodes of non-bloody non-bilious emesis with associated abdominal pain. Hemoglobin down-trended from 10.3/dL to 5.7 g/dL. Further laboratory investigations are displayed in Table 2.

**Table 2:** Additional hematologic studies

	Result	Reference Range
Haptoglobin	<10 mg/dL	37-355 mg/dL
Total bilirubin	0.7 mg/dL	<1 mg/dL
Reticulocyte count	3.6%	<2%
Reticulocyte index	1.72	<2 = hypoproliferation
Direct antiglobulin test	Negative	Negative
ADAMTS13 activity	50.5%	>66.8% is normal

Repeat peripheral blood smear demonstrated moderate schistocytes. Urine studies revealed a 24-hour urine protein of 2.6 g/24 hours and a urine protein to creatinine ratio of 3.528 mg/g (Table 3). Autoimmune work-up showed C3 of 107 mg/dL (reference range: 90-180 mg/dL), C4 of 26 mg/dL (reference range: 10-40 mg/dL), negative antinuclear antibodies, <1 anti-dsDNA, negative anti-PLA2R, and negative THSD7A. The urine protein electrophoresis test (UPEP) was interpreted as mild selective glomerular nephropathy with no monoclonal protein present. The patient was diagnosed with nephrotic syndrome and microangiopathic hemolytic anemia, both likely drug-induced due to the time course with sunitinib administration, worsened in the setting of IV antibiotics. She was maintained on empiric IV methylprednisolone with a prolonged steroid taper as an outpatient. Her laboratory parameters improved throughout her hospital course and subsequently thereafter. Her jaw lymphadenitis completely resolved with the completion of an antibiotic course.

**Table 3:** Urinalysis

Urinalysis		Reference range
Color	Yellow	
Protein	≥500	Negative
Glucose	50	Negative
Blood	Moderate	Negative
RBC	30/hpf	0-3/hpf

As an outpatient, the patient completed a course of steroids. Her anasarca resolved but her lower extremity edema persisted. The patient was maintained off of sunitinib due to her history of adverse reactions. A staging CT scan showed the progression of the disease, so the patient was started on everolimus. Her lower extremity edema is being managed with oral diuretics.

## Discussion

Sunitinib is a TKI that inhibits angiogenesis by targeting vascular endothelial growth receptor factor (VEGF) and PDGF. Both VEGF and PDGF have been shown to play crucial roles in tumor angiogenesis, growth, and metastasis. Thus, VEGF inhibitors have been widely and successfully used as chemotherapeutic agents. Sunitinib has been approved for the treatment of numerous cancers, including renal cell carcinoma (RCC), imatinib-resistant gastrointestinal stromal tumors, and metastatic well-differentiated pNET. Commonly published side effects of sunitinib include diarrhea, hand-foot syndrome, cytopenias, and fatigue [4]. However, with increased use of sunitinib therapy, cases of proteinuria, nephrotic syndrome, and TMA have been identified.

Chen et al. described a case of nephrotic syndrome and acute renal failure, requiring hemodialysis, in an elderly patient being treated for metastatic RCC with sunitinib. They hypothesized that sunitinib amplifies the pathogenesis of minimal change nephropathy, as well as induces an immune reaction, which leads to nephrotic syndrome [3]. Biopsy-proven TMA was described in a 44-year-old female, who was being treated for malignant, refractory skin hidradenoma with sunitinib. The patient developed hypertension and proteinuria after two weeks of sunitinib and received a kidney biopsy because of persistent proteinuria, which demonstrated features typical of TMA, including endothelial swelling, focal glomerular capillary thrombosis, and mesangiolysis [5]. The addition of an angiotensin-receptor blocker, in this case, helped to control the patient’s hypertension and proteinuria. In a case series of patients treated with sunitinib or sorafenib, seven patients were noted to develop a preeclampsia-like syndrome, characterized by hypertension, proteinuria, and hypoalbuminemia, similar to the patient described in this case. However, there was no evidence of microangiopathic hemolytic anemia in the patients studied. A dramatic improvement in proteinuria and laboratory markers was noted with either cessation or dose reduction of TKIs [6].

There have been several hypotheses on the mechanisms of the effects of sunitinib on renal microvasculature. In an experimental study with mouse models, researchers deleted the VEGF gene from renal podocytes and mice who had a deletion of VEGF had pronounced proteinuria, hypertension, and increased urine albumin-to-creatinine ratio. Electron micrographs of the glomeruli taken from the mutant mice demonstrated features consistent with TMA. It was hypothesized that, in vitro, VEGF leads to the formation of fenestrations in the endothelium of glomerular cells, which promotes the development of microvascular injury [7].

The cause of renal injury and TMA in patients taking sunitinib is unclear but is hypothesized to possibly be due to poor renal reserve and coexisting prothrombotic states. A review of the published case reports of sunitinib-induced TMA demonstrates that the majority of patients had hypertension and nephrotic or sub-nephrotic proteinuria [8]. The majority of cases of TMA caused by sunitinib have been published in patients being treated for RCC, which further supports the theory that poor renal function may be a catalyst to the development of TMA. Our patient was being treated for pNET, indicating that these side effects can develop regardless of underlying malignancy.

## Conclusions

We describe a case of proteinuria, acute kidney injury, and TMA in a patient with pNET, who initially responded to sunitinib but after a treatment holiday, upon re-initiating sunitinib, developed laboratory abnormalities suggestive of nephrotic syndrome and microangiopathic hemolytic anemia. Prompt discontinuation of sunitinib and the addition of high-dose steroids led to the resolution of the patient’s anasarca, anemia, thrombocytopenia, and kidney dysfunction. Further investigations are needed to determine the best practices in cases such as these, as there currently is no expert consensus or scientific literature on the optimal management of TKI-induced nephrotic syndrome, including the optimal usage and dosage of steroids. While incidence is rare, TKIs will continue to be used in oncologic management for various cancers in the foreseeable future, so guidelines should be considered for such rare and potentially life-threatening complications.
